# An Indoor Mapping Algorithm Fusing LiDAR-IMU Tightly Coupled Fusion and Scan Context: IS-LEGO-LOAM

**DOI:** 10.3390/s26092789

**Published:** 2026-04-30

**Authors:** Junying Yun, Zhoufeng Liu, Xintong Wan, Gefei Duan, Bowen Tian, Yajing Gao

**Affiliations:** 1 School of Electronic and Electrical Engineering, Zhengzhou University of Science and Technology, No. 1 Xueyuan Road, Mazhai Industrial Park, Erqi District, Zhengzhou 450064, China; xintongwan@zit.edu.cn (X.W.); dgf147852@zit.edu.cn (G.D.); tianbowen116@126.com (B.T.); mailgyj@163.com (Y.G.); 2 School of Information and Communication Engineering, Zhongyuan University of Technology, No. 41 Zhongyuan Middle Road, Zhengzhou 450007, China; lzhoufeng62@163.com

**Keywords:** LEGO-LOAM, indoor environment, tightly coupled LiDAR-IMU, scan context

## Abstract

Indoor environments often contain numerous areas with sparse structural features, such as long corridors, large atriums, and glass curtain walls, and other scenarios. These conditions can lead to difficulties in loop closure detection and accumulated positioning errors, resulting in localization drift or even mapping failure during map construction. This paper proposes an indoor mapping algorithm called IS-LEGO-LOAM that integrates tightly coupled LiDAR-IMU fusion and Scan Context. A tightly coupled LiDAR-IMU odometry is constructed, and an adaptive covariance matrix is designed to solve the problems of abnormal LiDAR echoes and insufficient effective feature extraction caused by sparse indoor feature points. By introducing the Scan Context global descriptor and adopting the strategies of vector nearest neighbor search and similarity score matching, the drift problem in large-scale scenes is alleviated. Finally, validation is performed on the KITTI dataset and in real-world scenarios, respectively. Experiments show that the improved IS-LEGO-LOAM achieves superior mapping performance.

## 1. Introduction

**Figure 1:**
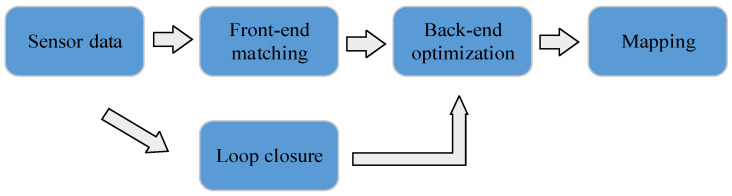
Schematic diagram of the SLAM framework.

With the continuous exploration and advancement of mobile robots in various application scenarios, their applications have become increasingly extensive and diverse. Simultaneous Localization and Mapping (SLAM) has been widely applied in the field of indoor robots. It is a technology that performs simultaneous localization and map construction in unknown environments using only onboard sensors, eliminating the need for pre-set base stations [1]. The SLAM framework is mainly composed of four components: front-end matching, back-end optimization, loop closure detection, and map construction [2]. Figure 1 presents a schematic diagram of the SLAM framework composition. According to the type of front-end sensors, SLAM can be categorized into visual SLAM with cameras as front-end sensors and LiDAR SLAM with LiDAR as front-end sensors [3]. Camera sensors are low-cost and can acquire abundant feature information; however, they cannot directly measure distance and are significantly susceptible to illumination variations, resulting in low precision [4]. In contrast, LiDAR is not affected by illumination, featuring fast response speed and extremely high ranging precision, yet it is relatively expensive [5,6]. With the advancement of science and technology and the increasing demand for LiDAR applications, the capability of mass production and popularization of LiDAR has been gradually improved, and LiDAR-based SLAM technology is developing at a rapid pace.

Currently, LiDAR SLAM technology can be divided into 2D LiDAR SLAM and 3D LiDAR SLAM [7]. The front-end sensor adopted in 2D LiDAR SLAM is a 2D LiDAR, which can only perceive single-plane information of the environment, and the maps constructed thereby are mostly two-dimensional. In a 3D LiDAR, multiple laser beams are arranged at a certain angle. By rotating a full circle through optical ranging, it can capture a massive number of laser points, which converge into a point cloud that reflects the real information of the surrounding environment. 3D LiDAR offers an intuitive mapping effect and high ranging precision; moreover, it is not affected by illumination changes and viewing angles in the environment during operation. Therefore, 3D LiDAR has become an indispensable sensor in map construction.

Currently, most 3D LiDAR SLAM algorithms with good map construction performance are applied in outdoor scenarios, and there is no dedicated 3D LiDAR SLAM algorithm specifically optimized for indoor scenarios. Indoor scenarios are different from outdoor scenarios in that they are structured environments. Compared with outdoor scenarios, indoor scenes have significantly fewer feature points, and featureless environments seriously impair the performance of LiDAR-based SLAM frameworks, leading to large drift or even map construction failure during indoor map construction. Therefore, this paper proposes an indoor-adaptable IS-LEGO-LOAM method. (1) A LiDAR-IMU tightly coupled odometer is constructed at the front-end of the algorithm, and an adaptive covariance matrix is designed to automatically adjust the number of degrees of freedom for feature matching, so as to solve the problems of abnormal LiDAR echoes and insufficient extraction of effective feature points; (2) The Scan Context global descriptor is introduced in the loop closure detection part of the algorithm, and a strategy of vector k-nearest neighbor search and similarity score matching is adopted to alleviate the drift problem in large-scale scenarios.

## 2. Related Work

LiDAR odometry relies on point cloud matching to achieve odometry calculation. Sparse point clouds, insufficient feature points, and structured environments such as long corridors can severely degrade the accuracy and robustness of LiDAR odometry [8]. Researchers have improved the accuracy and robustness of LiDAR odometry by fusing IMU data, which is referred to as LiDAR Inertial Odometry (LIO) [9]. The fusion of LiDAR and IMU is mainly realized through two approaches: filtering and optimization [10]. Representative filtering-based methods include LINS [11], FAST-LIO [12], FAST-LIO2 [13], LIO-GVM [14], and Point-LIO [15]. Centered on Kalman filtering and its variants, filtering-based methods incorporate IMU pre-integration and LiDAR matching errors into the state estimation framework to update pose and sensor noise in real time. They feature fast response and low computational overhead, yet are susceptible to model linearization errors. Optimization-based methods are founded on factor graphs or sliding windows, which jointly optimize constraints from multi-frame LiDAR-IMU data and reduce cumulative errors through global adjustment, thereby achieving higher localization accuracy. Nevertheless, they impose higher demands on computational resources and require a balance between real-time performance and optimization scale. Typical representatives of graph optimization include hdl_graph_slam [16], LIO-SAM [17], D-LIOM [18] and Spin-LOAM [19].

Loop closure detection is a core functional module of the SLAM system. Its key value lies in suppressing cumulative trajectory drift and optimizing map consistency through revisited scene recognition and the introduction of global constraints, making it a critical technology to support high-precision mapping in large-scale indoor environments [20]. At present, loop closure detection in LiDAR SLAM has formed a systematic research framework. Scholars worldwide have conducted in-depth research around three core objectives: robustness improvement, detection efficiency optimization, and scene adaptability expansion, giving rise to two mainstream technical paradigms: geometric feature matching-based methods and global descriptor-based methods [21,22]. Geometric feature matching-based methods focus on the geometric and topological information of point clouds and identify loop closure candidates through direct inter-frame feature matching. The theoretical foundation is laid by the Iterative Closest Point (ICP) algorithm [23] and its variants, such as the Normal Distribution Transform (NDT) [24]. However, such methods tend to fall into local optima in sparse-feature scenes. Global descriptor-based methods reduce the dependence on local features by encoding the overall structure of the scene and have thus become a research hotspot. Scan Context, first proposed by Korea’s KAIST University [25], constructs rotation-invariant representations through polar grid division and height information statistics. Kim G. et al. [26] later presented a spatially enhanced descriptor based on Scan Context, which improved the robustness of the algorithm against lateral and rotational variations and enhanced computational efficiency. Nevertheless, this method exhibits high computational complexity, making it difficult to apply in practical scenarios. In sparse or degraded environments, backend matching alone is insufficient to ensure robustness. Explicit treatment of feature reliability and dynamic/outlier removal is critical. Existing works [27,28] have demonstrated that semantic-aware filtering, dynamic feature rejection, and feature grading can effectively improve robustness, providing valuable guidance for reliable feature selection in complex environments.

## 3. Methodology

### 3.1. IMU-Fused I-LEGO-LOAM

**Figure 2:**
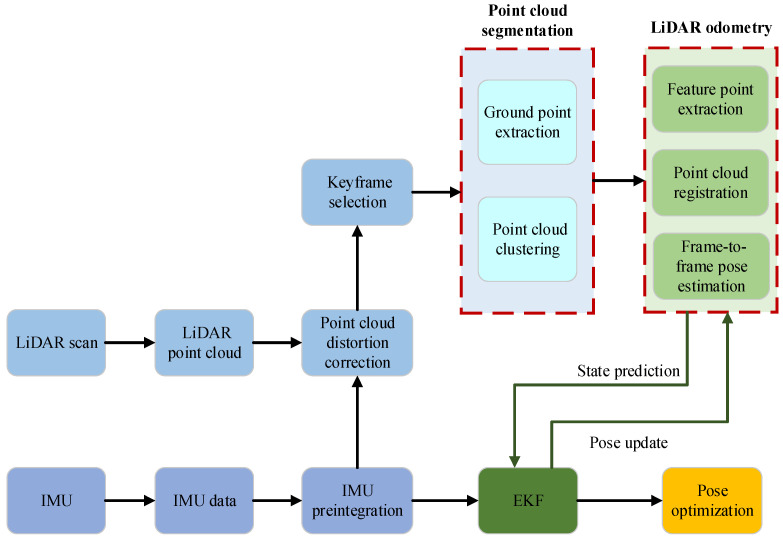
Schematic diagram of IMU-fused LEGO-LOAM.

A full scan of LiDAR yields a laser point cloud, from which pose information can be obtained through point cloud registration. IMU can provide corresponding velocity and position information based on data collected by accelerometers and gyroscopes. In this paper, IMU is fused into the system in two ways. First, it assists LiDAR in removing point cloud distortion. Second, it is applied to LiDAR odometry, where the Extended Kalman Filter (EKF) is adopted to fuse the pose information after registration and the real-time output of IMU to obtain more accurate pose estimates. This paper adopts a lightweight, tightly coupled architecture that combines IMU-assisted LiDAR distortion correction and EKF fusion, designed specifically for low-power embedded platforms in indoor environments. The schematic diagram of IMU-fused LEGO-LOAM is shown in Figure 2.

#### 3.1.1. IMU-Assisted LiDAR Distortion Correction

**Figure 3:**
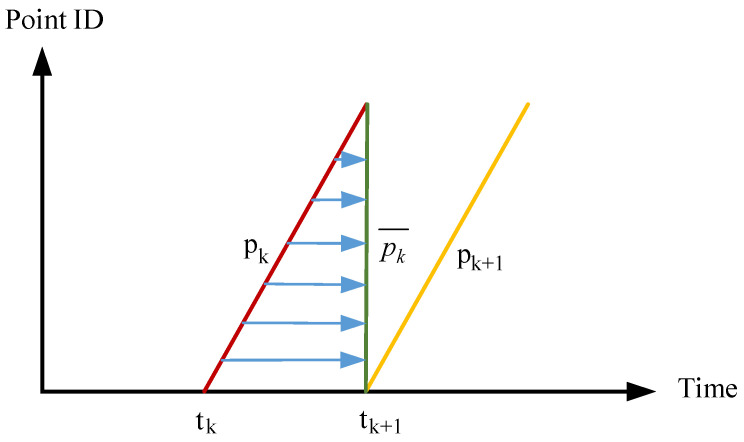
Schematic diagram of timestamp annotation for point clouds.

In practical applications of 3D LiDAR, a mobile robot equipped with LiDAR is usually in motion rather than stationary. Multiple laser beams rotate around a central axis to acquire environmental information. If the mobile robot moves during the scanning process, the starting scan point and the ending scan point of one frame of the 3D LiDAR point cloud will be inconsistent. This leads to different coordinate systems for each point in the point cloud, so that the point cloud cannot reflect the real information of the surrounding environment. This phenomenon is known as point cloud distortion. In this paper, an IMU-assisted method is employed to correct distorted point clouds. The acquired point cloud data are stamped with timestamps according to their acquisition sequence. The underlying principle is illustrated in Figure 3.

The horizontal axis, time, denotes the scanning time of the LiDAR, and PointID represents the frame ID of the point cloud. $t_{k}$ denotes the starting scan point of a LiDAR scan frame, and $t_{k+1}$ denotes the ending scan point of the same frame. The sampling frequency of the LiDAR is known and fixed, so the interval $t_{k+1}-t_{k}$ is also known and fixed. The sampling time of each point in a point cloud frame can be obtained via IMU-based pose estimation, and each point can be projected to the same timestamp. The horizontal arrow indicates that all LiDAR points in one frame are projected to the moment $t_{k+1}$. $\bar{p_{k}}$ represents a full frame of point cloud acquired by LiDAR after a 360° rotation, where different LiDAR points are annotated with different timestamps. Each LiDAR point is corrected according to the correction transformation matrix $T_{\left(k+1,i\right)}^{L}$. The correction matrix is given in Equation (1).

(1) $$T_{\left(k+1,i\right)}^{L}=\frac{t_{i}-t_{k+1}}{t_{k}-t_{k+1}}T_{k+1}^{L}$$

The coordinate of each original laser point is multiplied by the correction transformation in Equation (1) to obtain the coordinate information of the corrected laser point.

#### 3.1.2. Adaptive Covariance Matrix

The robot covariance matrix $Q_{L}$ is a matrix used to characterize the measurement errors and uncertainties of LiDAR. It includes positional errors of the LiDAR in the X, Y, and Z directions, as well as roll, pitch, and yaw errors. The magnitudes of these errors significantly affect the performance and accuracy of the LiDAR odometry algorithm. In the LEGO-LOAM algorithm, the values of the covariance matrix can be set manually or obtained through experimental measurements. Proper configuration of the covariance matrix improves the accuracy and stability of LiDAR odometry, whereas excessively large or small covariance values may lead to algorithm instability or degraded accuracy. The covariance matrix is usually a diagonal matrix, whose form is given in Equation (2).

(2) $$Q_{L}=diag(g_{x},g_{y},g_{z},g_{\phi },g_{\theta },g_{\psi })$$

where $g_{x}$, $g_{y}$, $g_{z}$, $g_{\phi }$, $g_{\theta }$, $g_{\psi }$ are constant values determined by experimental tests.

The $\xi (n_{e},n_{p})$ function determines the number of degrees of freedom (DOFs) to be retained in scan matching. Here, $n_{e}$ denotes the number of edge feature points matched, and $n_{p}$ denotes the number of planar points matched. The value of the $\xi (n_{e},n_{p})$ function determines the number of available degrees of freedom for matching. During the matching process, a small value of ξ indicates that only part of the degrees of freedom of the point cloud are matched, whereas a large value of ξ means more degrees of freedom are used, yielding more accurate matching results. Nevertheless, a large value of ξ also increases the computational burden. The $\xi (n_{e},n_{p})$ function is expressed in Equation (3).

(3) $$\xi (n_{e},n_{p})=(\frac{n_{me}-\min(n_{me},n_{e})}{n_{me}})(\frac{n_{mp}-\min(n_{mp},n_{p})}{n_{mp}})+\xi_{\min}$$

As the number of features decreases, the error in pose estimation from the SLAM system increases. In this paper, the covariance matrix in LiDAR odometry is given by

(4) $$Q_{L}=diag(g_{x},g_{y},g_{z},g_{\phi },g_{\theta },g_{\psi })\xi (n_{e},n_{p})$$

They are statistically measured through repeated experiments on the KITTI dataset and in indoor environments, ensuring stable system operation under normal conditions. $g_{x}$, $g_{y}$, $g_{z}$, $g_{\phi }$, $g_{\theta }$, $g_{\psi }$ are adjusted to $g_{x}=4.7\times 10^{-3}$, $g_{y}=2.1\times 10^{-3}$, $g_{z}=1.5\times 10^{-3}$, $g_{\phi }=4.4\times 10^{-3}$, $g_{\theta }=5.2\times 10^{-3}$, $g_{\psi }=5.2\times 10^{-3}$. The lower bound $\xi_{\min}$ of $\xi (n_{e},n_{p})$ is set to a minimum value of 0.005. When $n_{e}$, $n_{p}$ approaches 0, $\xi (n_{e},n_{p})$ approaches its maximum value $1+\xi_{\min}$; when $n_{e}$ and $n_{p}$ approach $n_{me}$ and $n_{mp}$ respectively, $\xi (n_{e},n_{p})$ approaches its minimum value $\xi_{\min}$.

It should be clarified that the proposed adaptive covariance matrix is not a simple matching confidence adjustment merely based on the number of edge and planar features. Instead, it performs online uncertainty modeling for observation noise within the state estimation framework of tightly coupled LiDAR-IMU odometry. Unlike naive posterior confidence weighting using feature counts, our method directly maps feature abundance to observation uncertainty and integrates it into the noise model for optimization and filtering, enabling the estimator to perceive observation quality changes, thus differing fundamentally in theoretical formulation and working mechanism. The adopted function has explicit bounds, monotonicity, and high computational efficiency, ensuring numerical stability and real-time performance, making it a reasonable and effective choice for indoor sparse-feature scenarios. Meanwhile, this strategy aligns with the widely adopted quality-aware weighting paradigm in recent SLAM research. Adham et al. [29] proposed adaptive covariance based on alignment reliability to handle geometric degeneracy in urban environments, while Mansour et al. [30] employed adaptive quality gating for robust heading estimation under low-quality observations; both adapt constraint reliability dynamically according to observation quality. Our method follows this core idea, using the number of valid features to represent observation quality and adjusting uncertainty via adaptive covariance. It should be noted that this approach enhances robustness in weak-feature environments through global covariance scaling but does not explicitly model directional degeneracy, and thus cannot fully resolve the hallway problem, where longitudinal uncertainty increases significantly in long corridor scenarios. Direction-aware adaptive strategies will be explored in future work to more precisely handle anisotropic observation degeneracy.

### 3.2. Loop Closure Detection with Scan Context Fusion

**Figure 4:**
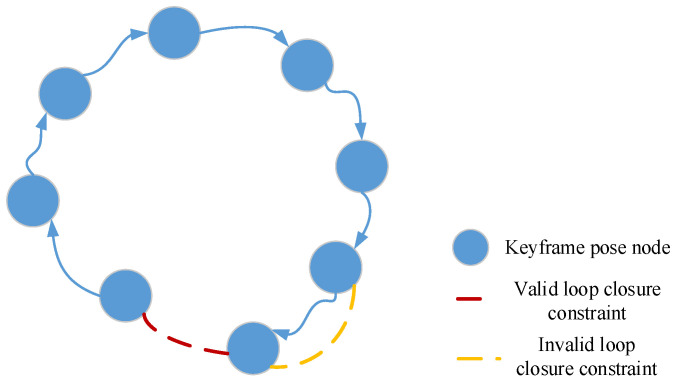
Invalid loop between consecutive frames.

LiDAR operates at a sampling frequency of 10 Hz, and the difference between two consecutive point cloud frames is relatively small. Including every scanned frame in loop closure detection would significantly slow down the algorithm. Therefore, this paper selects a certain number of data frames as keyframes based on time intervals. If the time interval for keyframe selection is too large, the number of keyframes will be insufficient to accurately represent the real environment. Conversely, if the interval is too short, especially during indoor mapping where obstacles are dense and the robot moves slowly, the physical distance between the robot positions corresponding to two consecutive frames becomes small. In such cases, the system is highly likely to establish a loop closure detection relationship between the current point cloud frame and the previous one. As shown in Figure 4, this kind of loop closure corresponds to an invalid loop.

**Figure 5:**
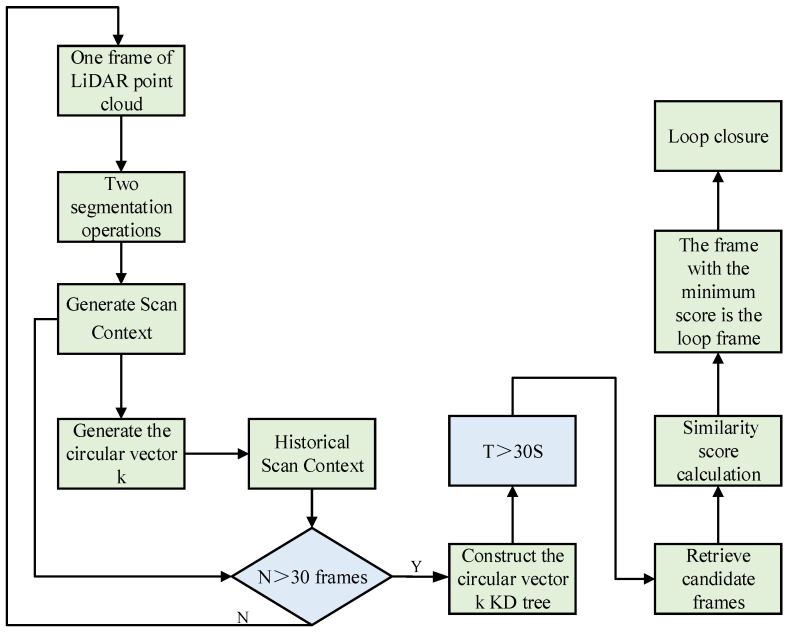
Loop closure detection with Scan Context global descriptor.

To avoid such invalid loop closure detection, this chapter sets a frame count threshold and a time threshold, respectively, and the specific procedure is shown in Figure 5.

When a new laser point cloud frame is received, the frame is segmented twice to generate the Scan Context global descriptor, which is then stored in the history descriptor container. The ring vector k is computed using Equations (5) and (6). A threshold of 30 frames is set for the number of descriptors in the history descriptor container. If the number of history descriptors is less than 30, new point cloud frames continue to be added. If the number exceeds 30, the point cloud interval between the selected frames must be greater than 30, and a KD-tree based on the ring vector k is constructed. A time threshold is applied before KD-tree nearest neighbor search, requiring the time difference T between the current frame and the historical frame to be greater than 30 s. Finally, a two-step nearest neighbor search algorithm is used to find similar frames, and the similarity scores between the current frame and these candidates are calculated. The frame with the minimum similarity score is selected as the loop frame for loop closure detection. Through the above frame count and time threshold settings, invalid loops between consecutive frames can be effectively avoided.

(5) $$\psi (r_{i})=\frac{||r_{i}|{|}_{0}}{N_{s}}$$

(6) $$k=(\psi (r_{1}),\ldots ,\psi (r_{N_{r}}))$$

where $r_{i}$ denotes a row in the Scan Context descriptor, and $N_{s}$ represents the number of sectors.

## 4. Experiment and Analysis

### 4.1. Experimental Environment

(1) Dataset Testing Environment

The algorithm proposed in this paper is tested on the KITTI 07 dataset. The KITTI dataset is used to verify the accuracy improvement of the proposed algorithm over the baseline method, not to demonstrate its performance in indoor scenarios. The processor used in the experiment is the 12th Gen Intel(R) Core(TM) i9-12900H, the graphics card model is GeForce RTX 3060 Laptop GPU, the operating system is Ubuntu 18.04, and the corresponding ROS version is ROS Melodic. The LiDAR experimental parameters used in the experiment are listed in Table 1.

The experimental parameter settings are shown in Table 1. Among them, N_SCAN denotes the number of LiDAR lines, Horizon_SCAN denotes the number of sampling points per full rotation of the LiDAR; ang_res_x and ang_res_y represent the horizontal and vertical angular resolutions of the LiDAR, respectively; ang_bottom denotes the angle between the LiDAR and the horizontal direction; groundScanInd indicates the LiDAR line index corresponding to ground points.

The EVO evaluation tool is used for quantitative testing. The commands evo_ape tum tum_07_gt.txt I-lego_loam.txt -r full -va --plot --plot_mode xyz and evo_ape tum tum_07_gt.txt IS_lego_loam.txt -r full -va --plot --plot_mode xyz are adopted to evaluate the absolute pose error during mapping. Details of the evo_ape parameters are listed in Table 2.

(2) Real-World Scene Testing Environment

**Figure 6:**
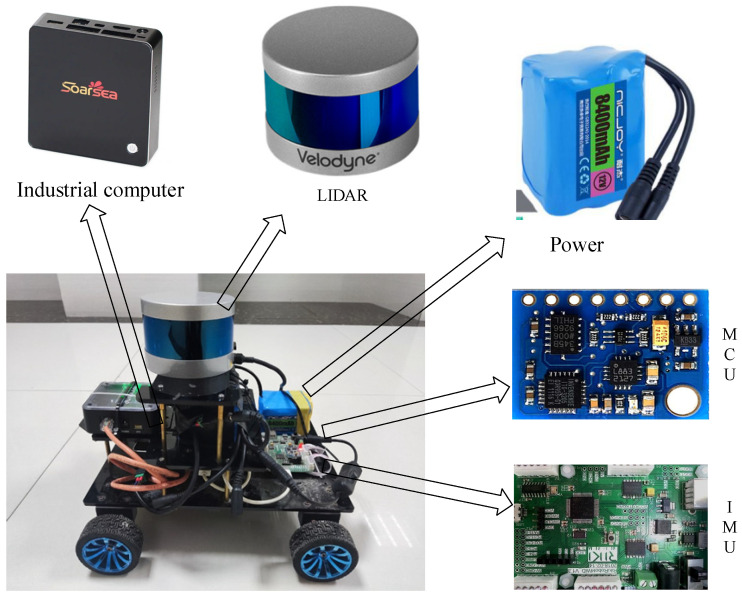
Mobile robot platform.

To compare LEGO-LOAM and IS-LEGO-LOAM in real-world scenarios, a mobile robot platform based on a 3D LiDAR sensor is built in this paper, which can realize the laser SLAM function. In the mobile robot system, the 3D LiDAR and IMU are responsible for collecting external environmental information and sending it to the industrial computer for processing. The MCU is in charge of driving the DC motors and sensors as well as performing corresponding data processing. The robot platform is shown in Figure 6.

While the robot navigates inside the building, it mostly travels back and forth along corridors in addition to moving through doors. In this paper, the 555 Studio in the Engineering Building and the long corridor on the fifth floor of the Engineering Building are selected as the experimental environments.

① Indoor Scene

**Figure 7:**
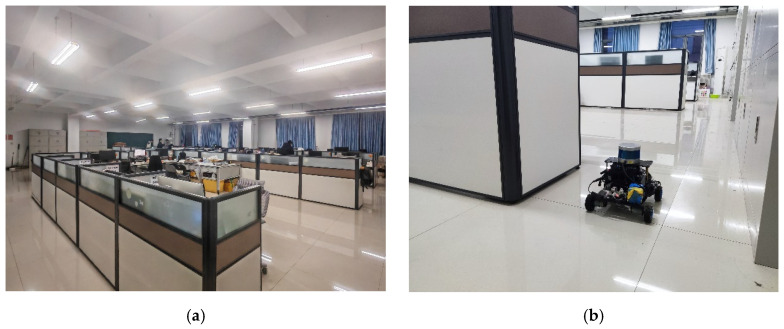
Studio environment and mapping process: (**a**) studio environment; (**b**) mapping process.

The overall environment of Studio 555 in the Engineering Building is shown in Figure 7a. The studio is 16 m long and 14.68 m wide, with workstations distributed in the middle, cabinets occasionally placed against the surrounding walls, and crisscrossing beams and columns overhead. A laptop is used to control the robot to construct a map by walking around the studio once, and the mapping process of the robot is shown in Figure 7b.

② Large-Scale Corridor Scene

**Figure 8:**
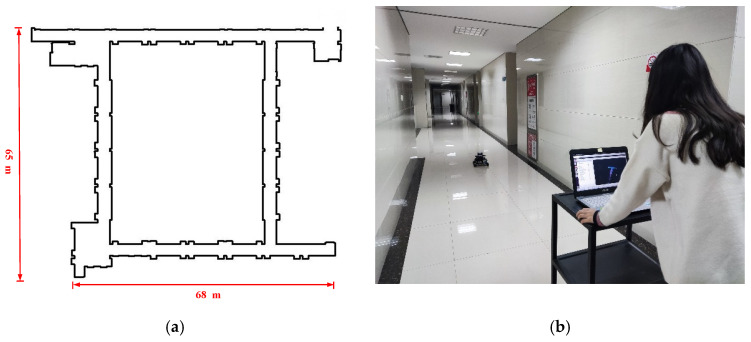
Actual mapping scene: (**a**) plan view of long corridor scene and (**b**) mapping process.

After the robot finishes mapping in the studio, a large-scale corridor environment is selected for testing. The large-scale corridor environment is illustrated in Figure 8a, which is generally rectangular. The environment has a complex structure with a long distance and sparse environmental features, and contains many similar scene structures, posing a great challenge to robot localization and mapping. The corridor scene is 68 m long and 65 m wide, with inconsistent corridor widths in different sections. The robot is controlled to perform mapping in the long corridor environment, and the mapping process is shown in Figure 8b.

### 4.2. Test Results and Analysis

#### 4.2.1. Dataset Environment Testing

(1) I-LEGO-LOAM with IMU Fusion

**Figure 9:**
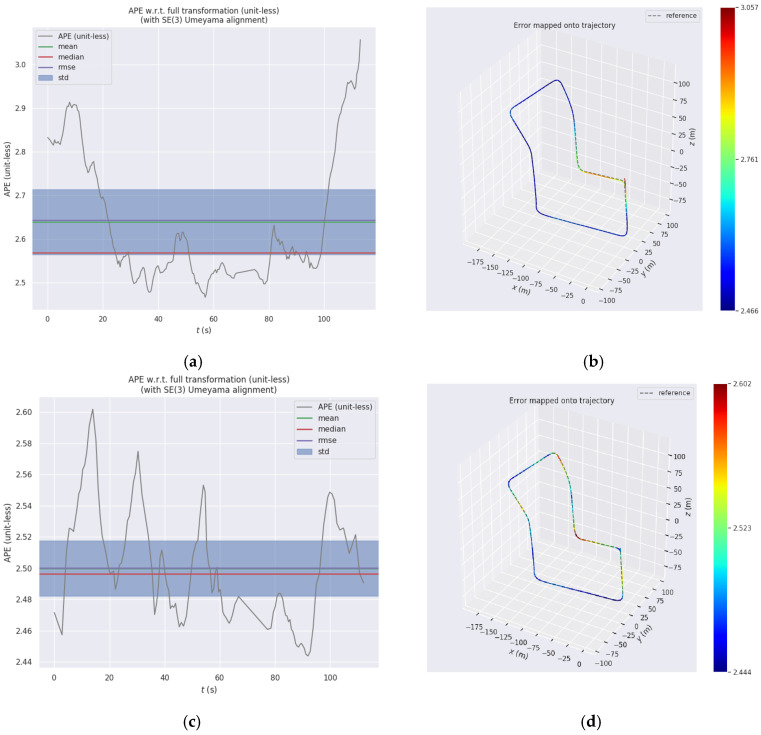
Comparison of absolute errors between LEGO-LOAM, I-LEGO-LOAM and ground truth: (**a**) APE values of LEGO-LOAM; (**b**) APE values of LEGO-LOAM trajectory; (**c**) APE values of I-LEGO-LOAM; and (**d**) APE values of I-LEGO-LOAM trajectory.

After evaluation using evo_ape, the absolute pose errors (APEs) between LEGO-LOAM, I-LEGO-LOAM and the ground truth are shown in Figure 9. Figure 9a depicts the absolute pose error of the LEGO-LOAM algorithm, and Figure 9c shows that of the I-LEGO-LOAM algorithm. Figure 9b,d illustrates the absolute pose error values corresponding to the trajectory maps of LEGO-LOAM and I-LEGO-LOAM, respectively. The dashed line represents the ground truth provided by the KITTI dataset, while the colored trajectories are the map trajectories generated by the algorithms. Different colors in the figures indicate different absolute pose error magnitudes, which increase gradually from blue to red. A detailed statistical table comparing the absolute errors of the LEGO-LOAM and I-LEGO-LOAM algorithms is presented in Table 3.

By comparing the mapping trajectories and absolute errors of the LEGO-LOAM algorithm and the IMU-fused I-LEGO-LOAM algorithm, it can be clearly seen from Figure 9 and Table 3 that the I-LEGO-LOAM algorithm with IMU fusion has a trajectory closer to the ground truth, and its mapping accuracy is also superior to the traditional LEGO-LOAM algorithm.

(2) IS-LEGO-LOAM with further Scan Context fusion

**Figure 10:**
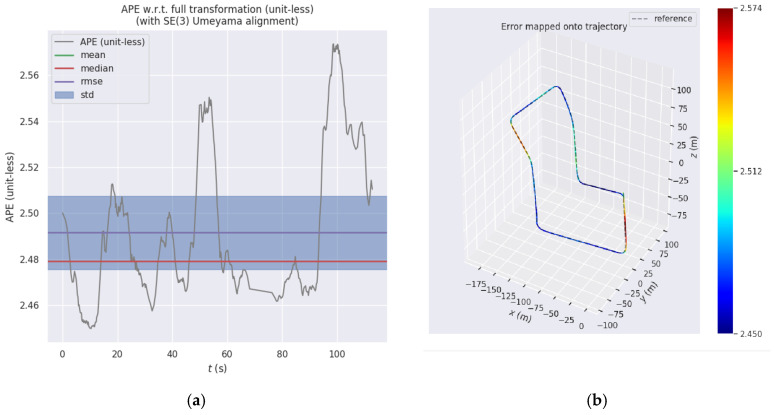
Absolute error comparison of IS-LEGO-LOAM: (**a**) APE values of IS-LEGO-LOAM and (**b**) APE values of IS-LEGO-LOAM trajectory.

Based on the I-LEGO-LOAM algorithm in the previous section, this section conducts comparative experiments on the absolute error among the IS-LEGO-LOAM algorithm with the Scan Context global descriptor introduced, the LEGO-LOAM algorithm, and the I-LEGO-LOAM algorithm. The evo plotting tool is used to draw the absolute error between IS-LEGO-LOAM and the ground truth. Figure 10 shows the absolute error between the IS-LEGO-LOAM algorithm and the ground truth, where Figure 10a presents the APE values of the IS-LEGO-LOAM algorithm, and Figure 10b shows the APE values of the IS-LEGO-LOAM algorithm trajectory. Table 4 is the comparison table of absolute errors among the LEGO-LOAM algorithm, I-LEGO-LOAM algorithm, and IS-LEGO-LOAM algorithm.

Through the above experimental comparisons, it can be clearly seen from Table 3 and Table 4 that after integrating IMU into the LEGO-LOAM algorithm, all performance indicators of the I-LEGO-LOAM algorithm are improved. With the further introduction of the Scan Context global descriptor, the IS-LEGO-LOAM algorithm achieves additional performance enhancements. Comparative verification of the improved LEGO-LOAM algorithms on the dataset shows that the enhanced IS-LEGO-LOAM algorithm outperforms the traditional LEGO-LOAM algorithm in all aspects, among which the standard deviation is reduced by 78.65% compared with LEGO-LOAM.

#### 4.2.2. Real Scene Testing

(1) Indoor Scene

**Figure 11:**
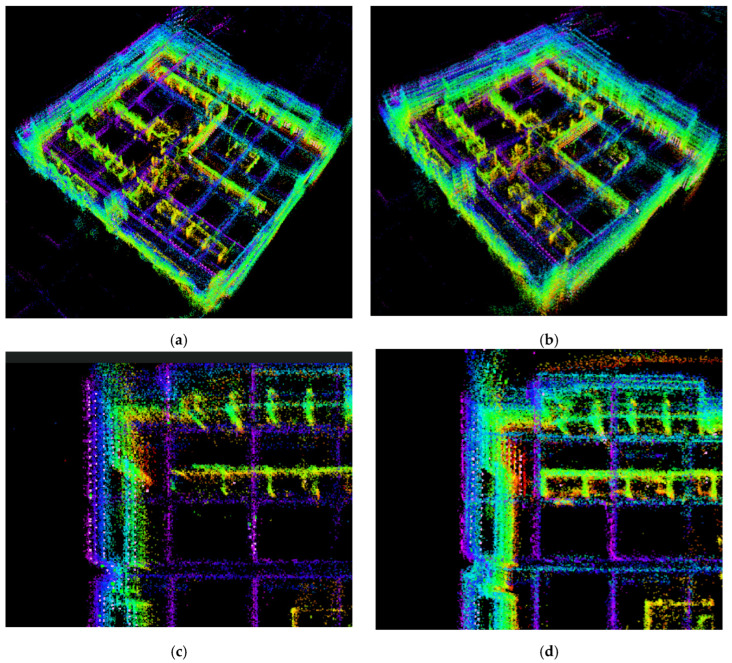
Mapping effect diagrams of LEGO-LOAM and IS-LEGO-LOAM: (**a**) overall mapping effect of LEGO-LOAM; (**b**) overall mapping effect of IS-LEGO-LOAM; (**c**) mapping details of LEGO-LOAM; and (**d**) mapping details of IS-LEGO-LOAM.

Figure 11a,c shows the mapping results of the LEGO-LOAM algorithm, while Figure 11b,d shows the mapping results of the IS-LEGO-LOAM algorithm.

The studio scene is small, and the mapping time is short. As can be clearly seen from Figure 11a,b, both the LEGO-LOAM and IS-LEGO-LOAM algorithms can construct a complete map. By comparing the map details in Figure 11c,d, it can be observed that the workstation mapping is incomplete and the point cloud map is relatively sparse when using the LEGO-LOAM algorithm. In contrast, the IS-LEGO-LOAM algorithm can generate complete workstations with a relatively dense point cloud map.

(2) Large-Scale Corridor Scene

**Figure 12:**
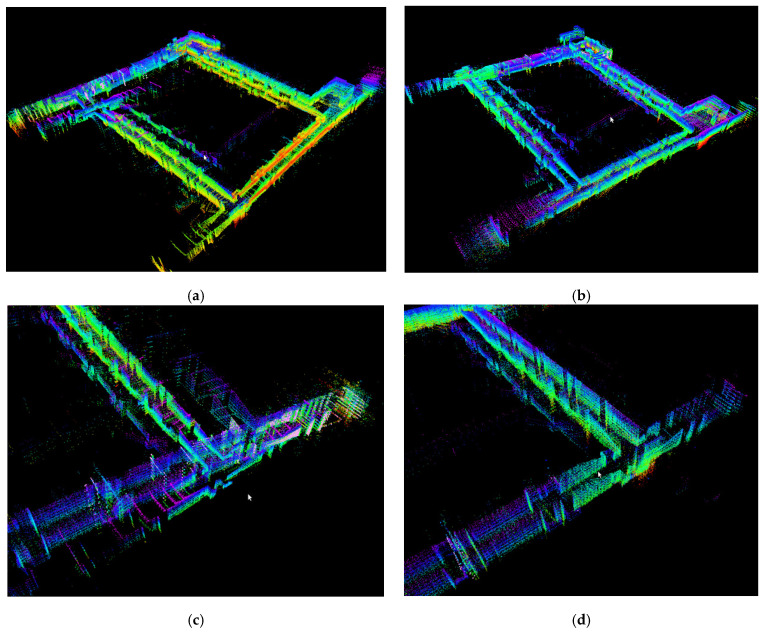
Detailed comparison of maps constructed before and after LEGO-LOAM improvement: (**a**) overall mapping effect of LEGO-LOAM in the long corridor; (**b**) overall mapping effect of IS-LEGO-LOAM in the long corridor; (**c**) mapping details of LEGO-LOAM; and (**d**) mapping details of IS-LEGO-LOAM.

The map constructed by the LEGO-LOAM algorithm is shown in Figure 12a, and the map constructed by the IS-LEGO-LOAM algorithm is shown in Figure 12b. Figure 12c,d shows the detailed comparison of the maps built by LEGO-LOAM and IS-LEGO-LOAM, respectively.

**Figure 13:**
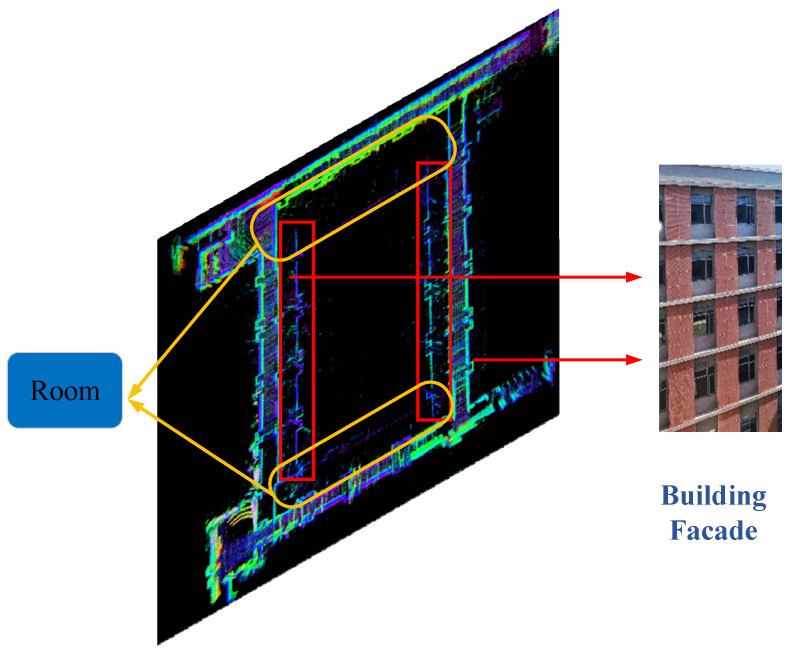
Map detail explanation.

The robot conducts mapping in a long corridor. Taking the top view in Figure 13 as an example, the left and right sides correspond to the building facades, as indicated by the red boxes in Figure 13. The upper and lower yellow boxes represent rooms, so the robot does not produce the rough edge phenomenon on the upper and lower corridors as it does on the left and right sides. The maximum measurement distance of the LiDAR is 150 m, and faint laser beams can be seen around the upper and lower rooms when the robot passes through the left and right corridors.

It can be clearly seen from the comparison between Figure 12 and Figure 13 that the robot exhibits drift during mapping using the LEGO-LOAM algorithm in long corridor scenes with similar structures and insufficient features, resulting in mapping failure. In the same environment, the robot using the IS-LEGO-LOAM algorithm can construct a complete map without drift or malfunction. Experiments show that the IS-LEGO-LOAM algorithm has significant advantages over the LEGO-LOAM algorithm.

## 5. Conclusions

This paper presents an IS-LEGO-LOAM indoor mapping method that integrates tightly coupled LiDAR-IMU fusion and Scan Context. It aims to address the problems of localization drift and map failure during mapping caused by insufficient effective feature points extracted in environments with sparse indoor structural features, such as corridors, large atriums, and glass curtain walls.

At the front end of the algorithm, IMU is tightly fused, and an adaptive covariance matrix is used, which alleviates the problem of insufficient valid features in sparse indoor structural environments. Meanwhile, the Scan Context global descriptor is introduced in the loop closure detection module, with frame count and time thresholds set to alleviate drift in large-scale scenes.

Comparisons among LEGO-LOAM, I-LEGO-LOAM, and IS-LEGO-LOAM on the KITTI dataset show that the improved IS-LEGO-LOAM outperforms the conventional LEGO-LOAM in all error metrics. Finally, comparative experiments between IS-LEGO-LOAM and LEGO-LOAM are conducted in an indoor studio and a large-scale corridor environment, respectively. Experimental results demonstrate that IS-LEGO-LOAM has significant advantages over LEGO-LOAM, with its standard deviation reduced by 78.65% compared with the original algorithm.

It should be noted that this paper mainly focuses on indoor sparse features, structural degeneracy, and LiDAR abnormal echo scenarios, and does not involve dynamic object interference or dynamic feature filtering. Recent studies have shown that in sparse or degraded environments, system robustness cannot be guaranteed by backend optimization alone, and explicit modeling of feature reliability, as well as outlier feature filtering, are essential. In future work, we will integrate feature grading and dynamic awareness mechanisms to further improve the system’s robustness in complex indoor environments.

In addition, limited by the current experimental platform and dataset conditions, refined indoor quantitative comparisons have not been carried out. In future work, a dedicated indoor experimental platform will be built, and standard datasets covering typical scenes such as weak textures and long corridors will be collected to conduct comprehensive quantitative tests and ablation analyses, so as to further verify the effectiveness and robustness of the proposed method in indoor environments.

## Figures and Tables

**Table 1:** LiDAR experimental parameter settings.

Parameter	KITTI Dataset	Velodyne 16
N_SCAN	64	16
Horizon_SCAN	1024	1800
ang_res_x	360.0/float(Horizon_SCAN)	0.2
ang_res_y	33.2/float(N_SCAN-1)	2.0
ang_bottom	16.6 + 0.1	15.0 + 0.1
groundScanInd	15	7

**Table 2:** evo_ape parameters.

Parameter	Meaning
-r full	APE obtained by considering both rotation and translation errors, unit-less.
va	v: Output information related to the file data. a: Perform trajectory registration.
--plot	Plot.
save_plot	Generated images.
save_results	Save calculation results.

**Table 3:** Comparison of absolute pose errors between LEGO-LOAM and I-LEGO-LOAM.

APE	Error Metrics	LEGO-LOAM	I-LEGO-LOAM
Max	Maximum error	3.0571	2.6019
Mean	Mean error	2.6498	2.5000
Median	Median error	2.5683	2.4962
Min	Minimum error	2.4659	2.4437
RMSE	Root mean square error	2.6530	2.5003
STD	Standard deviation	0.1494	0.0353

**Table 4:** Error Comparison of LEGO-LOAM, I-LEGO-LOAM and IS-LEGO-LOAM.

APE	Error Metrics	LEGO-LOAM	I-LEGO-LOAM	IS-LEGO-LOAM
Max	Maximum error	3.0571	2.6019	**2.5735**
Mean	Mean error	2.6498	2.5000	**2.4915**
Median	Median error	2.5683	2.4962	**2.4793**
Min	Minimum error	2.4659	2.4437	**2.4499**
RMSE	Root mean square error	2.6530	2.5003	**2.4917**
STD	Standard deviation	0.1494	0.0353	**0.0319**

## Data Availability

Dataset available on request from the authors.

## Abbreviations

The following abbreviations are used in this manuscript:

SLAM	Simultaneous Localization and Mapping
LIO	LiDAR Inertial Odometry
NDT	Normal Distribution Transform
ICP	Iterative Closest Point
EKF	Extended Kalman Filter
